# Frontal recess anatomy in Japanese subjects and its effect on the development of frontal sinusitis: computed tomography analysis

**DOI:** 10.1186/s40463-015-0074-6

**Published:** 2015-05-29

**Authors:** Kazunori Kubota, Sachio Takeno, Katsuhiro Hirakawa

**Affiliations:** Department of Otolaryngology, Head and Neck Surgery, Division of Clinical Medical Science, Programs for Applied Biomedicine, Graduate School of Biomedical Sciences, Hiroshima University, Hiroshima, Japan; Department of Otorhinolaryngology, Hiroshima University School of Medicine, 1-2-3 Kasumi, Minami-ku, Hiroshima, 734-8551 Japan

**Keywords:** Frontal recess cells, Frontal recess anatomy, Frontal sinusitis

## Abstract

**Background:**

Comprehensive understanding of frontal recess anatomy is essential for the successful treatment of patients with frontal sinus disease. This study was designed to determine the prevalence of specific frontal recess cells in Japanese subjects and the association of these cells with the development of frontal sinusitis.

**Methods:**

Frontal recess anatomy was analyzed using high-resolution spiral computed tomography images of paranasal sinuses from December 2008 through September 2011. The distribution of various frontal recess cells in patients with and without frontal sinusitis was compared by logistic regression analysis.

**Results:**

A total of 150 patients met the criteria, and 300 sides were analyzed. Agger nasi cells were present in 88.0 % of sides; frontal cell types 1 (FC1), 2 (FC2), 3 (FC3), and 4 (FC4) were present in 37.0 %, 6.3 %, 4.3 %, and 1.3 %, respectively; supraorbital ethmoid cells in 6.0 %, suprabullar cells in 37.0 %, frontal bullar cells (FBC) in 7.0 %, and interfrontal sinus septal cells in 8.6 %. Multiple logistic regression analysis showed that the presence of FBCs was significantly associated with the development of frontal sinusitis (*p* = 0.043).

**Conclusions:**

The frequencies of frontal recess cells in Japanese adult patients were similar to those reported for other East Asian adult populations, including Chinese, Korean, and Taiwanese. Anatomically, FBCs may show a greater association with the development of frontal sinusitis than other frontal recess cells.

## Background

**Fig. 1:**
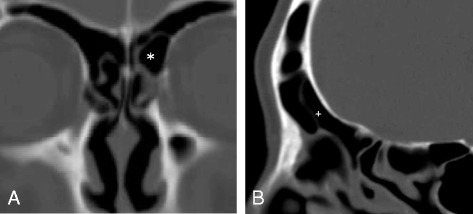
Computed tomography (CT) images of a healthy frontal sinus. **a** Coronal CT showing a left frontal cell type 3 (FC3) (*). **b** Sagittal CT showing a frontal bullar cell (FBC) (+)

**Fig. 2:**
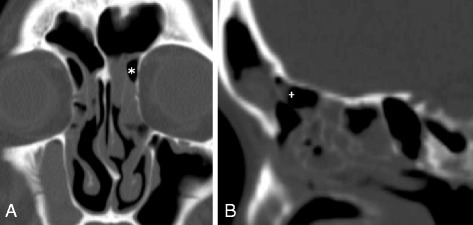
CT images of a patient with frontal sinusitis. **a** Coronal CT showing a left FC3 (*). **b** Sagittal CT showing an FBC (+)

Advances in endoscopic visualization and high-resolution computed tomography (CT) have enhanced the understanding of frontal recess anatomy. Before frontal sinus surgery, the variable frontal recess cells in each patient must be analyzed to plan a strategy for dissecting all cells disturbing the nasofrontal recess, including drainage of the frontal sinus. Frontal recess cells consist of a combination of cells, including agger nasi cells (ANCs), frontal cell types (FCs) 1 to 4, suprabullar cells (SBCs), supraorbital ethmoid cells (SOECs), frontal bullar cells (FBCs), and intersinus septal cells (IFSSCs) [1]. In healthy persons, the FC3 and FBC extend into the frontal sinus and narrow the nasofrontal recess, defined as the pathway draining the frontal sinus (Fig. 1). In patients with frontal sinusitis, the FC3 is positioned next to the orbit, and the FBC lies along the skull base (Fig. 2). Because of the complexity of the frontal recess and the risk during surgery of injuring the orbit and skull base, a comprehensive understanding of frontal recess anatomy is essential for treating frontal sinus disease successfully.

Although CT has been used to assess frontal recess pneumatization patterns [1–7], few studies in English have focused on Japanese adult populations. This study used high-resolution CT images to analyze Japanese patients with frontal sinusitis. We hypothesized that Stammberger’s theory may apply to the development of frontal sinusitis, and paid particular attention to FC3s, FC4s, SBCs, SOECs, and FBCs, which can narrow the ventilation pathway of the frontal sinus. The purpose of this study was to clarify the association of various frontal recess cells with the development of frontal sinusitis in Japanese patients by determining cell frequency in those with and without frontal sinusitis.

## Methods

The study was performed at the Department of Otorhinolaryngology–Head and Neck Surgery, Hiroshima University Hospital, Hiroshima, Japan. Between December 2008 and September 2011, 150 consecutive patients underwent CT of the nasal cavities and paranasal sinuses. Spiral CT scans of the nasal cavities and paranasal sinuses were performed on a Toshiba Aquilion CT scanner (Toshiba Medical Systems, Tokyo, Japan) with 1-mm-thick axial cuts. The following scanning parameters were used: kV 120, mA 200, window level 2000, central level 500. The CT data were reconstructed into coronal and sagittal images at a computer workstation. Imaging angles, contrast, and brightness were adjusted on the computer workstation to improve bony detail, which was especially useful for identifying severely diseased frontal recess cells in patients with rhinosinusitis.

**Fig. 3:**
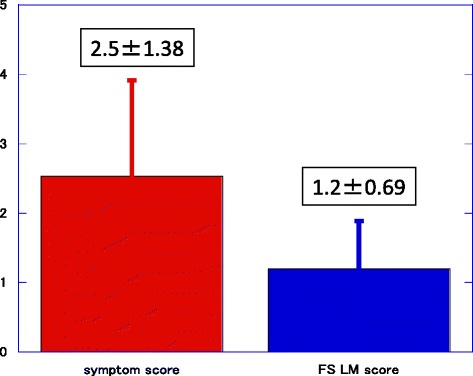
Symptom and Lund–Mackay scores of patients with frontal sinusitis. Symptom score was based on the SNOT-22. FS LM score: frontal sinus Lund–Mackay score

The 150 patients consisted of 50 patients with chronic rhinosinusitis and 100 controls, including 50 patients with allergic rhinitis without chronic rhinosinusitis and 50 normal individuals with no nasal symptoms. Exclusion criteria included previous sinus surgery, age <18 years, maxillofacial fracture, and/or sinonasal malignancy. CT images on which it was difficult to identify the delicate structures of the frontal sinus because of excessive motion or beam hardening artifacts were also excluded. Frontal sinusitis was defined as mucosal thickening >3 mm involving the entire frontal sinus or its dependent portions and the presence of symptoms. Fullness or heaviness of the frontal head, frontal pain, and 15 other sinonasal symptoms were also evaluated using the modified Sino-Nasal Outcome Test-22 scoring system, with scores ranging from 0 to 5; the average scores of fullness or heaviness of the frontal head and of frontal pain symptoms are shown in Fig. 3 as representative of frontal sinusitis patients.

The 300 sides of the 150 patients were categorized into three groups, based on the findings of frontal sinusitis on CT images and the presence of chronic rhinosinusitis (Table 1). Images were evaluated for the presence of ANCs, FCs, SBCs, SOECs, FBCs, and IFSSCs. FC types were determined according to modifications of previous criteria [1], which clarified the definitions of several types of frontal recess cells. The Lund–Mackay score of each paranasal sinus shadow was evaluated in all patients and compared in patients with and without frontal sinusitis [8]. To minimize variations in interpretation, each CT scan was evaluated jointly by two trained ear–nose–throat (ENT) surgeons, with any disagreements resolved by consensus.

**Table 1:** Data for 150 patients undergoing computed tomography

	CRS(+)		CRS(−)	Total
	Frontal sinusitis (+)	Frontal sinusitis (−)		
Age (years ± SEM)	57.8 ± 13.8	41.3 ± 17.5	47.0 ± 19.4	51.1 ± 18.4
Distinguishable sides	70	30	200	300
Male: Female	50:20	14:16	100:100	164:136
Lund-Mackay score (±SEM)	7.9 ± 2.4	5.5 ± 1.8	0.19 ± 0.64	2.6 ± 3.6
Anterior ethmoid score (±SEM)	1.8 ± 0.45	1.4 ± 0.55	0.025 ± 0.16	0.58 ± 0.84

CRS = chronic rhinosinusitis; SEM = standard error of the mean

Statistical analyses were performed using Excel Statistics 2010 (Shakai Jouhou Corp. Tokyo, Japan). Multivariate logistic regression analyses were performed to identify factors associated with frontal sinusitis. The odds ratio (OR) and 95 % confidence interval (CI) were calculated for each factor. ANCs, FC1–FC4s, SBCs, SOECs, FBCs, and IFSSCs were chosen as predictive variables. A *p* value <0.05 was considered statistically significant for all measurements. The protocol was approved by ethical committee of epidemiological research at Hiroshima University (No. 1063).

## Results

A total of 300 sides from 150 patients were assessed (Table 1). Seventy sides, 50 from men and 20 from women, showed evidence of frontal sinusitis, whereas 230 sides did not. The mean symptom score of patients with frontal sinusitis was 2.5 ± 1.38 and the Lund–Mackay score of their frontal sinuses was 1.2 ± 0.69 (Fig. 3). Fourteen patients (14 sides) had unilateral frontal sinusitis and 28 (56 sides) had bilateral frontal sinusitis. Patients with frontal sinusitis were older (57.8 ± 13.8 years) than those without frontal sinusitis (41.3 ± 17.5 years), but the difference was not significant (*p* = 0.469). Mean Lund–Mackay score (7.9 ± 2.4 vs. 5.5 ± 1.8, *p* = 0.000) and mean anterior ethmoid score (1.8 ± 0.45 vs. 1.4 ± 0.55, *P* = 0.001) were significantly higher in patients with than without frontal sinusitis.

**Fig. 4:**
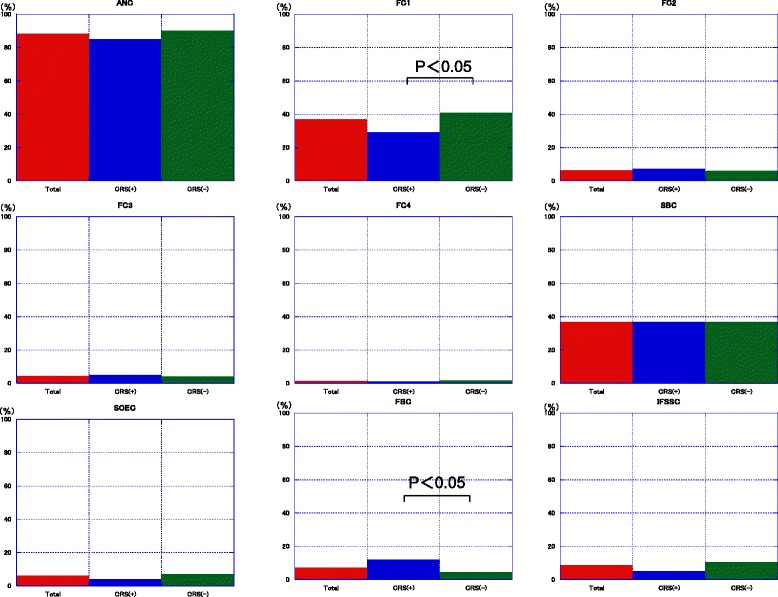
Percentages of various frontal recess cells identified on CT images. ANC: agger nasi cells; FC1–FC4: frontal cell types 1–4; SBC: suprabullar cells; SOEC: supraorbital ethmoid cells; FBC: frontal bullar cells; IFSSC: intersinus septal cells. CRS(+): patients with chronic rhinosinusitis; CRS(–): patients without chronic rhinosinusitis; Total: all patients with and without chronic rhinosinusitis

In categorizing frontal recess cells, we found ANCs in 265 sides (88.0 %) and FCs in 147 (49.0 %), with FC1s in 111 sides (37.0 %), FC2s in 19 sides (6.3 %), FC3s in 13 sides (4.3 %), and FC4s in four sides (1.3 %). SBCs, SOECs, FBCs, and IFSSCs were observed in 111 (37.0 %), 18 (6.0 %), 21 (7.0 %), and 26 (8.6 %) sides, respectively (Table 2). In comparing the percentage of frontal recess cells in patients with [CRS(+)] and without [CRS(–)] chronic sinusitis (Fig. 4), we found a significant difference in the presence of FC1s. In addition, the presence of FBCs was significantly higher in the CRS(+) than in the CRS(–) group. There was no difference in the distribution of frontal recess cells between normal controls and patients with allergic rhinitis. Multivariate analysis showed that the presence of FBCs was strongly associated with an increased frequency of frontal sinusitis (*p* = 0.043) (Table 3).

**Table 2:** Incidence of frontal recess cells in various populations

Cell types	Our cases Japanese; 300 sides, no. (%)	Taiwanese; 363 sides, no. (%)	Chinese, 404 sides, no. (%)	Korean, 114 sides, no. (%)	Caucasian, 82 sides, no. (%)
ANC	265 (88.0)	323 (89.0)	380 (94.1)	107 (94.0)	71 (86.6)
FC1	111 (37.0)	78 (21.5)	98 (24.4)	26 (22.8)	29 (35.4)
FC2	19 (6.3)	38 (10.5)	28 (7.0)	16 (14.0)	17 (20.7)
FC3	13 (4.3)	28 (7.7)	33 (8.2)	9 (7.9)	7 (8.5)
FC4	4 (1.3)	0 (0)	0 (0)	0 (0)	0 (0)
SBC	111 (37.0)	142 (39.1)	148 (36.6)	45 (39.5)	9 (11.0)
SOEC	18 (6.0)	28 (7.7)	22 (5.4)	3 (2.6)	53 (64.6)
FBC	21 (7.0)	23 (6.3)	36 (9.0)	16 (14.0)	5 (6.1)
IFSSC	26 (8.6)	35 (9.6)	25 (12.4)	10 (8.8)	6 (7.3)

**Table 3:** Statistical analysis of the effect of various frontal recess cells on the development of frontal sinusitis

Variable	CRS (+) 100 sides, no. (%)		CRS (−) 200 sides, no. (%)	Univariate analysis			Multivariate analysis		
	FS (+)	FS (−)		OR	95 % CI	*P* value	OR	95 % CI	*P* value
FBC	9 (13)	3 (10)	9 (4.5)	2.63	1.06–6.52	0.038*	2.56	1.03–6.38	0.043*
ANC	60 (86)	26 (87)	179 (89.5)	0.75	0.34–1.64	0.469			
FC1	21 (30)	8 (27)	82 (41)	0.65	0.37–1.15	0.14			
FC2	5 (7.1)	2 (6.7)	12 (6)	1.16	0.40–3.35	0.779			
FC3	4 (5.7)	1 (3.3)	8 (4)	1.46	0.44–4.89	0.54			
FC4	2 (2.9)	0 (0)	2 (1)	3.29	0.46–23.8	0.238			
SBC	22 (31)	15 (50)	74 (37)	0.77	0.44–1.35	0.358			
SOEC	4 (5.7)	0 (0)	14 (7)	1.26	0.43–3.66	0.673			
IFSSC	3 (4.3)	2 (6.7)	21 (11)	0.40	0.12–1.36	0.14			

*FS* frontal sinusitis, *OR* odds ratio, *CI* confidence interval. Asterisk indicates significance at p < 0.05

## Discussion

The frontal recess is a complex space that resembles an inverted funnel or cone, with the apex at the frontal ostium. This space is filled by various anterior ethmoid or frontal recess cells [9]. Because of the intrinsic anatomic complexity of this narrow space, comprehensive knowledge of frontal recess anatomy is required prior to surgery.

In investigating the prelavence of frontal recess cells on CT images, we found that the prevalence of ANCs was 88.0 %, similar to previous findings [1–4]. Although we found that the prevalence of FC1s in Japanese patients was almost as high as in Caucasians, the prevalence of other frontal cells (FC2–FC4s), especially FC2s, was in line with findings in other Asian populations. FC4s are independent of the appearance of ANCs [1]. Previous studies have reported FC4s in 16 (2.1 %) of 768 subjects [5] and in 3 (3.1 %) of 98 frontal recesses [6], making FC4s quite rare among frontal recess cells. In our study, nearly half (48.9 %) of the Japanese subjects had frontal cells.

Similar to findings in other East Asian populations, SBCs were more frequent while SOECs were less frequent, in Japanese than in Caucasian patients [1–4]. Although the prevalence of these frontal recess cells in our study population was more consistent with those in Chinese, Korean, and Taiwanese populations than with those in Caucasians, the prevalence of FC1s (37.0 %) in Japanese patients was closer to that in Caucasians (35.4 %) than in Taiwanese (21.5 %), Chinese (24.4 %), and Korean (22.8 %) groups. The latter discrepancy may be due to racial differences between Japanese and other East Asian populations [3].

The pathophysiology of frontal sinusitis is associated with ventilation of the sinus via the sinus ostium. The size of the frontal sinus ostium is key to frontal sinus drainage. Generally, frontal recess cells and their inflammation can influence frontal sinus ventilation by narrowing the frontal sinus drainage pathway. Because frontal cells may be associated with frontal sinus inflammation, we assessed whether frontal recess cells were associated with frontal sinusitis in Japanese subjects.

The association between the presence of anterior frontal recess cells (ANCs and FC1–FC4s) and the development of frontal sinusitis is unclear. Enlargement of ANCs has been found to correlate with a decrease on CT in the anterioposterior size of the nasofrontal recess, involved in the frontal sinus drainage pathway. The association between a requirement for revision sinus surgery in patients with frontal sinusitis and agger nasi disease was highly statistically significant. Failure to address agger nasi disease can contribute to failure of the primary surgery [10]. An analysis of 768 coronal CT scans showed that the prevalence of frontal mucosal thickening was higher in individuals with frontal cells of any type than in individuals without frontal cells, with the prevalence of FC3 and FC4 differing significantly [5].

Another study, however, found no difference in the frequency of frontal sinusitis on sides with and without frontal cells [6]. Moreover, the incidence of frontal sinusitis was not increased in patients with persistent ANCs undergoing revision surgery, and the diameters and areas of the frontal isthmus were similar in sinuses with various types of frontal cells.

In assessing the frontal recess cells posterior and posterolateral to the frontal recess (FBCs, SBCs, SOECs), our multivariate analysis suggested that the prevalenc of FBCs was associated with the development of frontal sinusitis. FBCs are characterized by pneumatization along the skull base in the posterior frontal recess and extend through the frontal ostium into the true frontal sinus. FBCs are significantly associated with the narrow anteroposterior diameter of the frontal ostium and frontal recess [2]. In frontal recesses that have FBCs, this anatomic tendency may play a role in narrowing the frontal sinus drainage pathway, resulting in significant obstruction.

Although anatomic variations in the frontal recess are likely to play a role in frontal sinusitis, mucosal inflammatory processes are also likely to be an important etiologic factor [6, 11, 12]. Allergies, asthma, and tobacco smoking can affect the nasal mucosa and lead to poorer outcomes of functional endoscopic sinus surgery, despite meticulous handling of the frontal ostium mucosa and preservation of the natural outflow tract [13]. An evaluation of 289 frontal recesses at the time of revision surgery found that 193 (66.8 %) had mucosal edema or polyposis obstructing the frontal recess [11]. In the absence of anatomic reasons for obstruction, mucosal inflammatory disease in the frontal recess should be considered a medical rather than a surgical problem. Seven major factors were associated with frontal sinusitis: mucosal disease (67 %); retained ethmoid cells (53 %); lateralized middle turbinates (30 %); retained ANCs (13 %); scar tissue (12 %); retained frontal cells (8 %); and neo-osteogenesis (7 %), with most frontal recesses having more than one factor (average 1.6) [11]. Frontal sinusitis is therefore caused by multiple factors, including anatomic variations, mucosal inflammation, and sinonasal polyposis. Further investigations are needed to understand the effects of anatomic variants of frontal recess cells on frontal sinusitis.

## Conclusions

The frequencies of frontal recess cells in Japanese adult patients were similar to those reported for other East Asian adult populations, including Chinese, Korean, and Taiwanese patients. Frontal bullar cells may have more influence on the development of frontal sinusitis than other frontal recess cells.

### Consent

Written informed consent was obtained from the patient for the publication of this report and any accompanying images.
